# Supplementary material for: A release-and-capture mechanism generates an essential non-centrosomal microtubule array during tube budding
Source: Nat Commun. 2021 Jul 2;12:4096. doi: 10.1038/s41467-021-24332-0 (PMC8253823; doi:10.1038/s41467-021-24332-0)
Supplement: Supplementary file 1 — Supplementary Information [file 41467_2021_24332_MOESM1_ESM.pdf]

Supplementary Material:

A release-and-capture mechanism generates an essential non-centrosomal microtubule array during tube budding

Ghislain Gillard[1], Gemma Girdler[1,2] and Katja Röper[1]*

[1]MRC-Laboratory of Molecular Biology, Francis Crick Avenue, Cambridge Biomedical Campus, Cambridge CB2 0QH, UK
[2]current address: Wellcome-MRC Stem Cell Institute, Jeffrey Cheah Biomedical Centre, Cambridge Biomedical Campus, University of Cambridge, Puddicombe Way, Cambridge, CB2 0AW, UK

Supplementary Figure 1, related to Figure 1. Microtubule rearrangements in the early salivary gland placode.

Surface views of placodes showing that microtubules undergo a 90º rearrangement during cell constriction. At early stage 11, labeling for tyrosinated α-tubulin (magenta) shows a dense microtubule network lying parallel to the apical surface of the cells (**a-a''**). During mid stage 11 these microtubule bundles change orientation (**b-b''**) to run perpendicular to the apical surface as longitudinal bundles by late stage 11 (**c-c''**). Constricting apices are marked by *srcGFP* in green. The salivary gland placode is indicated by a dotted line. White boxes indicate areas magnified in middle and right panels, arrows point to apical parallel microtubules in **a'** and the end foci of longitudinal bundles in **c'**, asterisks point to the invaginating pit once it has formed in **b, c**.

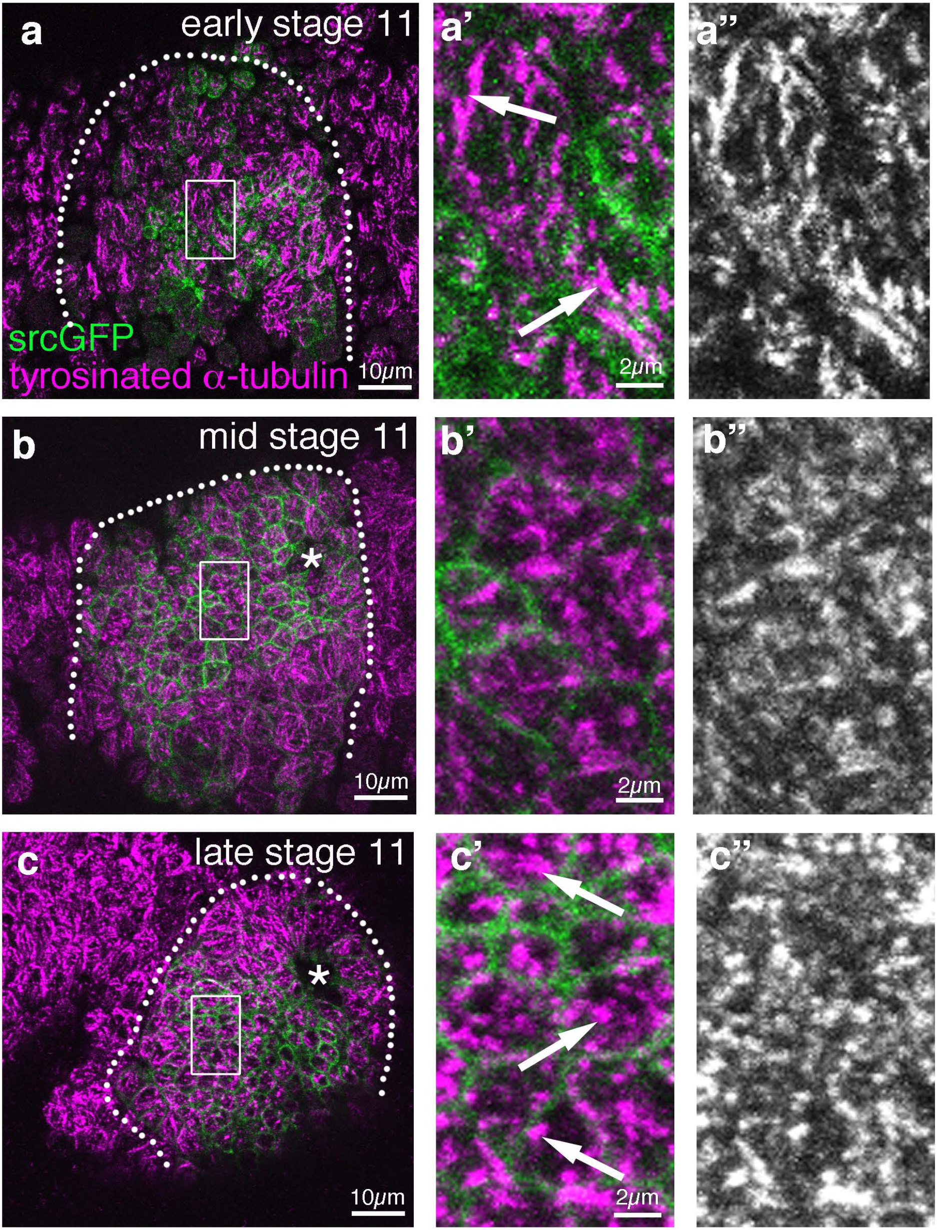

Gillard et al._Supplementary Figure 1, related to Figure 1

Supplementary Figure 2, related to Figure 1. Changes to centrosomes during post-blastoderm embryogenesis.

Comparison of Asl-mCherry (red, single channel in **a''-e''**) and Cnn (blue, single channel in **a'''-e'''**) levels at embryonic centrosomes at different stages and in different tissues. Cell outlines are labelled using Scribble-GFP that localises along the lateral membranes of all epithelial cells. Orange arrowheads point to centrosomes within a cell with even Cnn levels at both centrosomes, blue arrowheads point to asymmetric Cnn levels within a single cell. **a-a'''** During and just after cellularisation at stage 5, embryonic cells contain two centrosomes with even levels of Cnn. **b-b'''** During stage 8 in the lateral epidermis undergoing germ band extension, Cnn levels are even on both centrosomes and only strongly increased on centrosomes of cells about to undergo mitosis. **c-d'''** At stage 9 in the ventral (**c-c'''**) and dorsal (**d-d'''**) epidermis, cells with asymmetric Cnn levels can be found, likely representing cells in G2 of cycle 15 or 16. These cells will divide again and thus are on the way to generating two centrosomes with equal Cnn levels. **e-e'''** At stage 16 in the dorsal epidermis most centrosomes have lost all Cnn.

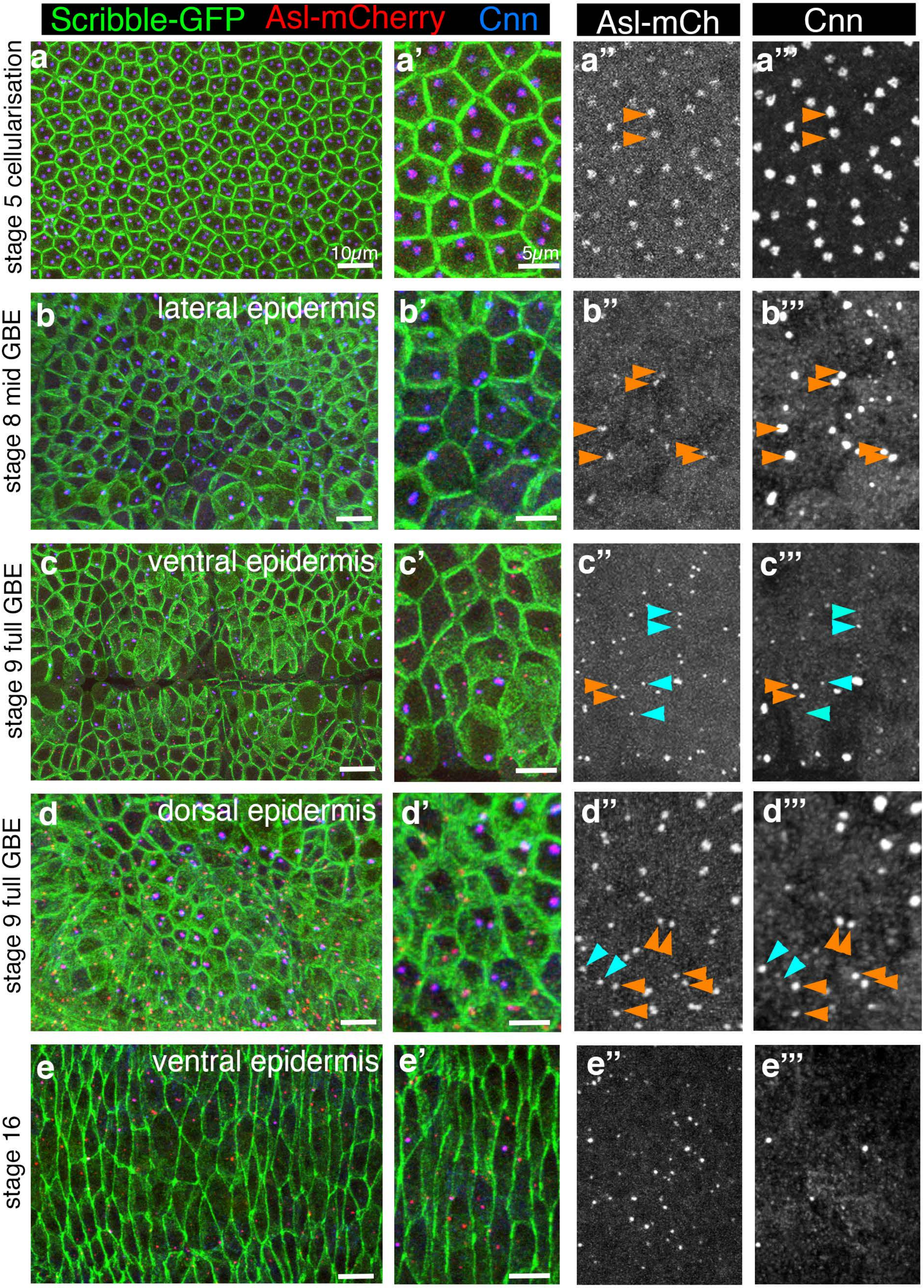

Gillard et al._Supplementary Figure 2, related to Figure 1

Supplementary Figure 3, related to Figure 2. Centrosome composition and nucleation asymmetry in placodal cells.

a-c Projected time series of *EB1-GFP Asl-mCherry* embryos in a region of a stage 11 salivary gland placode to reveal EB1-GFP 'comets' where they emanate from centrosomes. **b** shows a region with two Asl-mCherry centrosomes with EB1-GFP comets and thus actively nucleating microtubules, whereas **c** shows two Asl-mCherry centrosomes without comets and thus not nucleating.

d-d' Two further examples of centrosome asymmetries of Cnn and Cnb in individual placodal cells at stage 11, as shown in Fig. 2g.

e-g Comparative analysis of YFP-Centrobin (YFP-Cnb) and γ-tubulin asymmetries at centrosomes. **e-f'''** Overview (**e**) and higher magnifications (**f-f'''**) of a placode, with anti E-Cadherin in white, anti γ-tubulin in red, anti Asl in cyan and YFP-Cnb in green. Filled arrowheads in **f'-f'''** point to centrosomes with higher levels of γ-tubulin and YFP-Cnb enrichment, hollow arrowheads point to the centrosomes in the same cell with lower levels of γ-tubulin and YFP-Cnb. **g** Line scan profile through both centrosomes of a single cell to illustrate the co-enrichment of γ-tubulin and Cnb on the same centrosome.

h-j Comparative analysis of GFP-Polo and γ-tubulin asymmetries at centrosomes. **h-i'''** Overview (**h**) and higher magnifications (**i-i'''**) of a placode, with anti E-Cadherin in white, anti γ-tubulin in red, anti Asl in cyan and GFP-Polo in green. Filled arrowheads in **i'-i'''** point to centrosomes with higher levels of γ-tubulin and GFP-Polo enrichment, hollow arrowheads point to the centrosomes in the same cell with lower levels of γ-tubulin and GFP-Polo. **j** Line scan profile through both centrosomes of a single cell to illustrate the co-enrichment of γ-tubulin and Polo on the same centrosome.

The salivary gland placode is indicated by a dotted line and the invagination point by an asterisk.

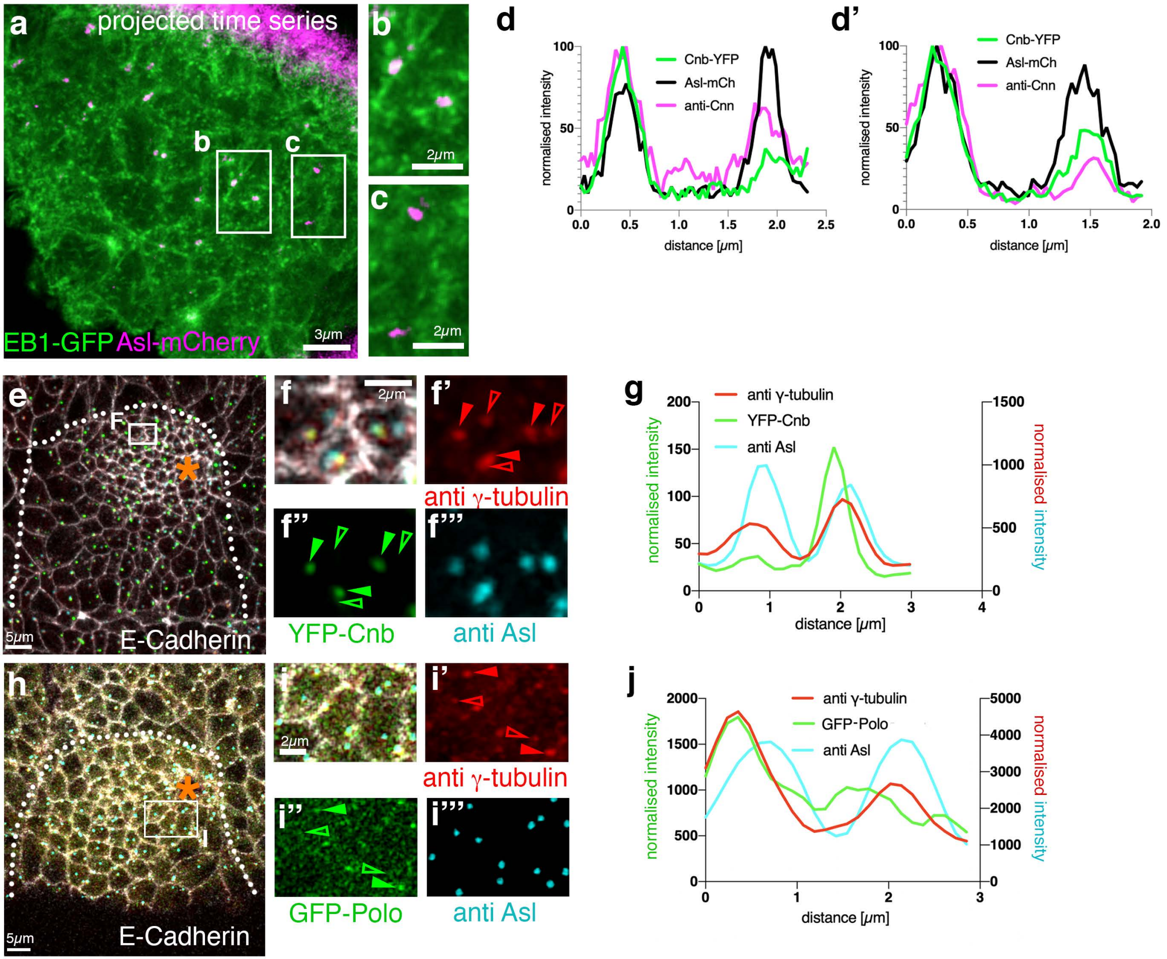

Gillard et al., Supplementary Figure 3, related to Figure 2

Supplementary Figure 4, related to Figures 3 and 4. γ-tubulin overexpression increases microtubule and actomyosin levels and leads to excessive apical constriction in the placode.

a-b' Stage 11 placode labelled for α-tubulin in a control (**a, a'**) or γ-tubulin overexpressing embryo *(***b, b'**; *ncd::γ-tubulin-EGFP*). E-Cadherin is in magenta.

c-d' Stage 11 placode visualising myosin using *sqh-RFP* in a control (**c, c'**) or γ-tubulin overexpressing embryo *(***d, d'**; *ncd::γ-tubulin-EGFP*). E-Cadherin is in magenta.

e Quantification of F-actin fluorescence in stage 11 placodes (as shown in Fig. 3f, f') in control embryos and γ-tubulin-EGFP overexpressing embryos, with 480 cells from 12 embryos quantified in the control and 520 cell from 13 embryos in *ncd::γ-tubulin-EGFP* embryos.. Data are shown as a violin plot with median and quartiles, statistical significance was determined by two-sided unpaired Mann-Whitney test as $p<0.0001$.

f-g' Overview of placodes shown in magnification panels in Figure 4h, i. In control placodes, cross-sections show that longitudinal microtubules at stage 11 are concentrated near the invagination pit (magenta bars in cross section panels in **f'**). By contrast, in γ-tubulin-EGFP overexpressing placodes longitudinal microtubules can be clearly found all throughout the placode (magenta bars in cross section panels in **g'**).

h-i'' Apical-medial Patronin-RFP, a stabiliser of free microtubule minus ends, accumulates excessively where increased numbers of microtubule bundle foci are found in placodal cells of *ncd::γ-tubulin-EGFP* embryos (**i-i''**) in comparison to control (**h-h''**). Composits corresponding to Figure 4 j, k are shown.

j-l'' In *ncd::γ-tubulin-EGFP* embryos placodal cells, more centrosomes nucleate microtubules than in control cells (**J**) , 231 centrosomes in 9 embryos were analysed in the control and 171 centrosome in 8 embryos in *ncd::γ-tubulin-EGFP* embryos; shown are mean +/- SD and statistical significance determined using two-sided unpaired t-test as $p=0.0002$.

k-l'' Time-projections of time-lapse movies were analysed to determine the amount of microtubule nucleation at centrosomes in control embryos of the genotype *Asl-GFP EB1-mCherry* and in embryos of the genotype *ncd::γ-tubulin-EGFP EB1-mCherry*. Shown are examples of projections of 10 consecutive time points for a control *Asl-GFP EB1-mCherry* placode (**k-k''**) and a *ncd::γ-tubulin-EGFP EB1-mCherry* placode (**l-l''**). Centrosomes are shown in **k, l**, EB1-mCherry to identify EB1 comets are shown in **k''**, **l''** and an overlay in **k'**, **l'** (centrosomes in magenta). Magenta arrowheads point to nucleating centrosomes and blue arrowheads point to non-nucleating centrosomes. See also Supplementary Movie 2.

The boundary of the placode is marked by a dotted line, and the invagination point is marked by an asterisk.

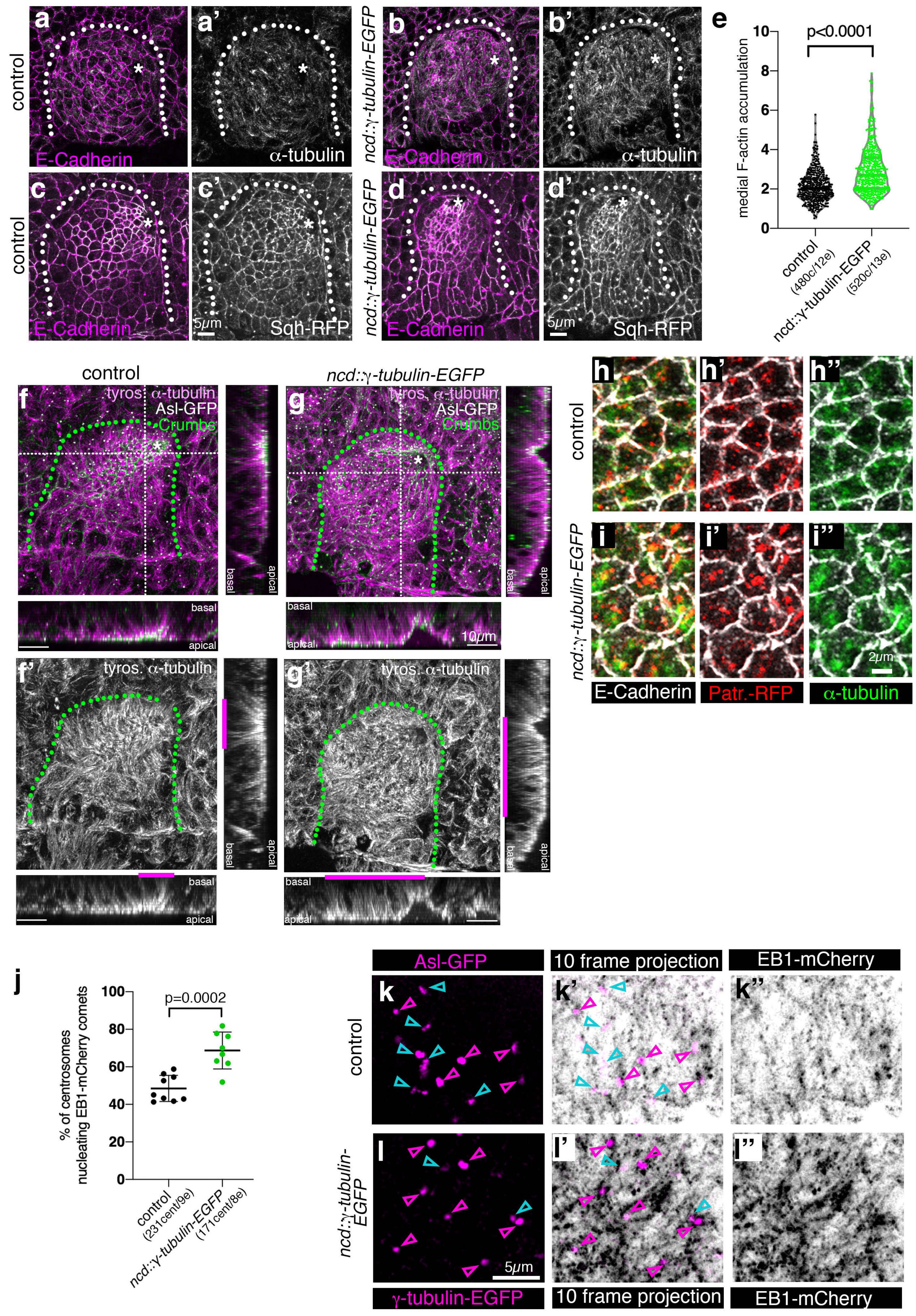

Gillard et al., Supplementary Figure 4, related to Figure 3 and 4

Supplementary Figure 5, related to Figure 7. Patronin localisation and dynamics in stage 10 -11 embryos.

a-b’ In a salivary gland placode at stage 10, prior to tissue bending and tube invagination commencing, Patronin-YFP localisation (green in **a**, single channel in **a’’** and **b**) is mostly restricted to adherens junctions marked by E-Cadherin (magenta in **a**, single channel in **a’** and **b’**).

c Quantification of medial Patronin localisation at stage 11, once tube invagination has commenced, in secretory cells in the placode compared to surrounding epidermal cells. 726 cells from 15 embryos in total were analysed for the placode and epidermis, shown are mean +/-SD, statistical significance was determined using two-sided unpaired Mann-Whitney test as $p<0.001$ (***).

d-f Live time-lapse analysis of Patronin-YFP; Ubi-TagRFP in a stage 11 placode in cells close to the invagination point (position of cells in **d, e** is indicated in **f** where the invagination pit is marked by an asterisk); Patronin-YFP is in green and Ubi-TagRFP in magenta. The time interval between frames (Δt) is 7.7 sec, the scale bars denote 5µm. Note the dynamic behaviour of Patronin-YFP.

g-i Depletion of Katanin80-YFP using *degradFP x fkhGal4* (**h-h’’**) leads to loss of apical-medial Patronin-RFP (green and **g’, h’**) in contrast to control (**g-g’’**). **g’’** and **h’’** are magnifications of the white boxes shown in **g** and **h**. Membranes are labelled by E-Cadherin (magenta). **I** Quantification of changes of the apical-medial vs apical-junctional Patronin-RFP localisation upon degradation of Katanin80-YFP (*Kat80-YFP degradFP ctrl:* 600 cells from 12 embryos; *Kat80-YFP degradFP fkhGal4:* 595 cells from 12 embryos; shown are mean +/-SD, statistical significance was determined by two-sided unpaired Mann-Whitney test as $p<0.0001$).

j-l Laser-ablation induced microtubule-severing of spindle microtubule in dividing cells leads to fast Patronin-RFP recruitment compared to control. Spindles were ablated in the same experiment as microtubules in early and late placodes, hence the control and early and late placode values shown are the same as in Fig. 7. **j** Time-resolved quantification of the fluorescence intensity of Patronin-RFP at the site of laser-ablation in spindles compared to control. Mean +/- SEM are shown, n=13 (spindle), n=22 (control). **k** Quantification of Patronin-RFP intensity in the first image acquired post-ablation in spindles and control. Shown are mean +/- SD are shown, n= 17 (early placodes), n=22 (late placodes), n=22 (control), n=13 (spindle); statistical significance was determined by two-sided unpaired Mann-Whitney test as $p<0.0001$ where indicated. **l** Laser-induced microtubule ablation. Compared to a control cut just below the apical microtubules (Fig. 7i), laser-induced ablation of microtubules in spindles (**l**) leads to a very rapid recruitment of Patronin-RFP to the newly generated minus-ends of microtubules. Orange arrowheads point to the severed site and

Patronin-RFP recruitment. A *Jupiter-GFP Patronin-RFP* genotype was analysed. The dotted boxes indicate the area shown in individual black and white panels. See also Supplementary Movies 3-6.

In overview panels the salivary gland placode is indicated by a dotted line and the invagination point by an asterisk.

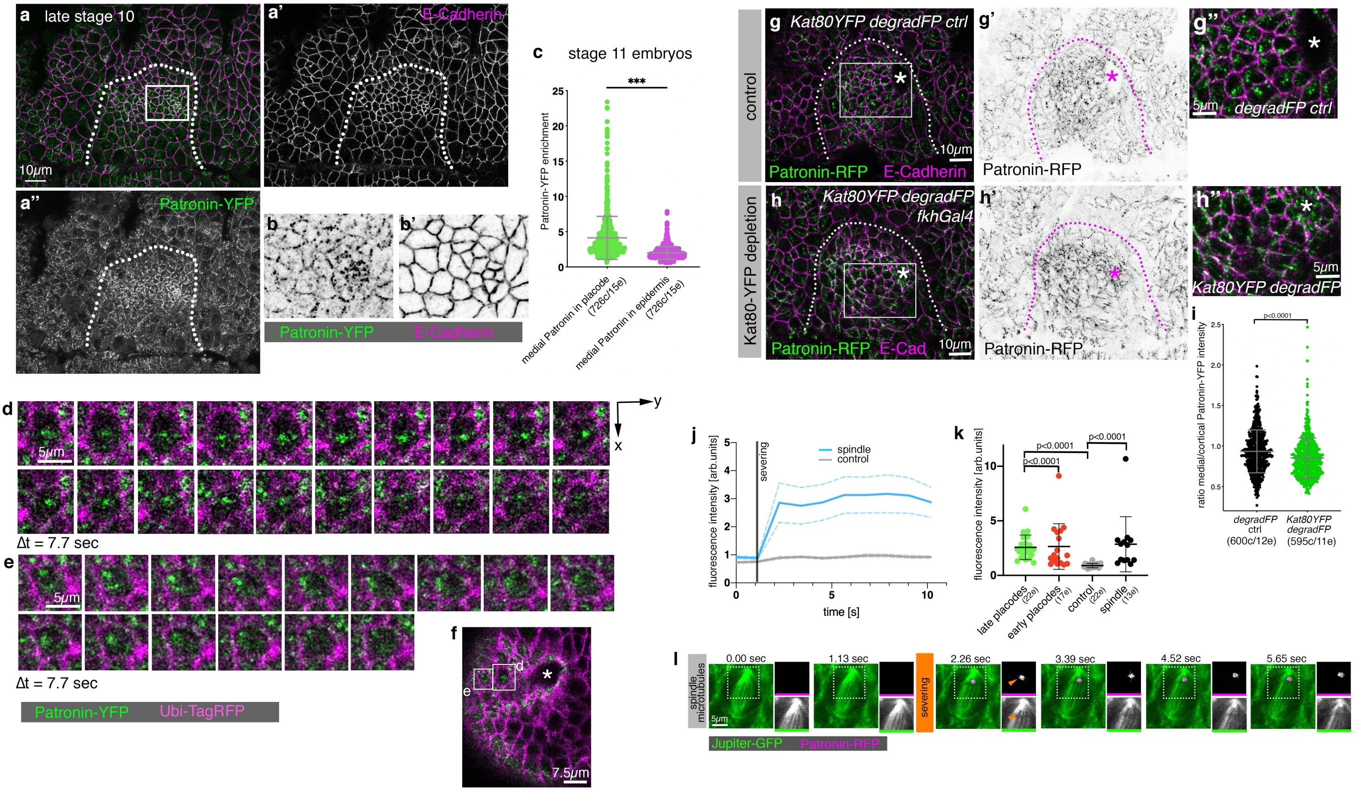

Gillard et al._Supplementary Figure 5, related to Figure 7

Supplementary Figure 6, related to Figure 8. Patronin depletion and its effects.
a-b'' Attempted Patronin depletion using degradation of Patronin-YFP via the degradFP system. In control embryos (**a-a''**) Patronin is localised to junctions and the apical-medial region within the placode and to junctional regions in the surrounding epidermis (magenta bracket in **a'**). In contrast in embryos of the genotype *Patronin-YFP; UAS-degradFP x Patronin-YFP; DaGal4* (**b-b''**) most junctional Patronin-YFP has been degraded (bracket in **b'**), but much apical-medial Patronin-YFP is left in the placode and also at times in select locations in the surrounding epidermis where Patronin colocalises with acetylated α-tubulin that remains (arrows in **b', b''**).
c, d Quantification of medial (**c**) and junctional (**d**) Patronin fluorescence intensity in control and *UAS-Patronin-RNAi x DaGal4* embryos. 250 cells from 7 placodes were analysed in the control and 214 cells from 5 placodes for *UAS-Patronin-RNAi x DaGal4* embryos; shown are mean +/- SD, statistical significance was determined by two-sided unpaired Mann-Whitney test as $p<0.0001$.
e Quantification of microtubule fluorescence intensity in placodal cells in control embryos and *UAS-Patronin-RNAi x DaGal4* embryos. Shown are mean +/-SD, note the absence of a significant difference as established by two-sided unpaired Mann-Whitney test as $p=0.3943$.
f-g''' Patronin-RFP colocalises with a pool of Katanin80-YFP in cells near the invagination pit. The boxed area in **f** is highlighted in **g-g'''**, arrowheads point to colocalisation.
Placode boundaries are indicated by dotted lines and the invagination point by an asterisk.
h-h'' Patronin-YFP (green) in the apical-medial region of secretory cells colocalises with the cytolinker Shot (Red). Cell outlines are labeled by E-Cadherin, arrowheads point to apical-medial foci where Shot and Patronin colocalise.
i-k Depletion of Patronin-YFP (green) using RNAi in *UAS-RNAi-Patronin x DaGal4* embryos (**j, j'**) leads to loss of apical-medial Shot labelling (red) in contrast to control (**i, i'**). **i'** and **j'** are magnifications of the white boxes shown in **i** and **j**. Membranes are labelled by E-Cadherin (white). **k** Quantification of changes of apical-medial Shot accumulation upon Patronin depletion (*ctrl:* 180 cells from 6 embryos; *UAS-RNAi-Patronin x DaGal4:* 150 cells from 5 embryos; shown are mean +/- SD, statistical significance was determined by two-sided unpaired Mann-Whitney test as $p<0.0001$).
In overview panels the salivary gland placode is indicated by a dotted line and the invagination point by an asterisk.

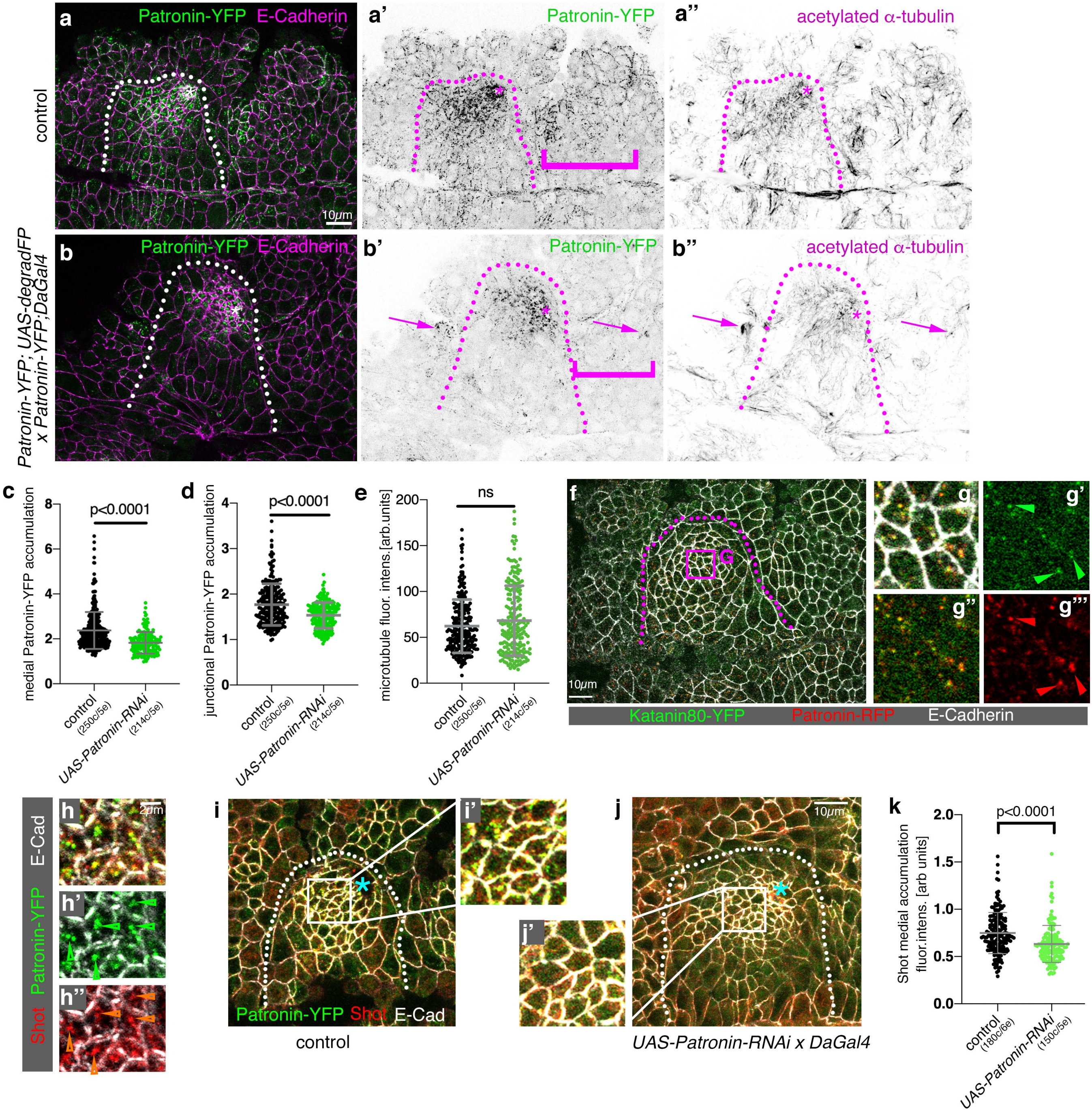

Gillard et al._Supplementary Figure 6, related to Figure 8

Supplementary Figure 7, related to Figures 3, 6, 8. Late stage gland phenotypes.

a-f' Gland phenotypes observed at later stages (stage 14/15) compared to control (**a-c**) in *ncd::γ-tubulin-EGFP* expressing embryos (**d-f'**).

g-l' Gland phenotypes observed at later stages (stage 14/15) compared to control (**g-i**) in *Katanin80-YFP fkhGal4 UAS-deGradFP* expressing embryos (**j-l'**).

m-r' Gland phenotypes observed at later stages (stage 14/15) compared to control (**m-o**) in *PatroninYFP DaGl4 UAS-Patronin-RNAi* expressing embryos (**p-r'**).

s Quantification of aberrant gland phenotypes observed in *ncd::γ-tubulin-EGFP, Katanin80-YFP fkhGal4 UAS-deGradFP* and *PatroninYFP DaGl4 UAS-Patronin-RNAi* and matching controls.

Orientation of views is indicated in each panel (lateral or dorsal). All embryos were labelled for E-Cadherin to show apical junctions and hence lumen shape of the glands. The basal side of the salivary gland tube epithelial cells is outlined in magenta. The aberrant part of lumens in experimental situations is marked by a box and amplified in a separate panel below. 'Rotated' views show the aberrant portion from a different angle, generated using 3D rendering in Imaris.

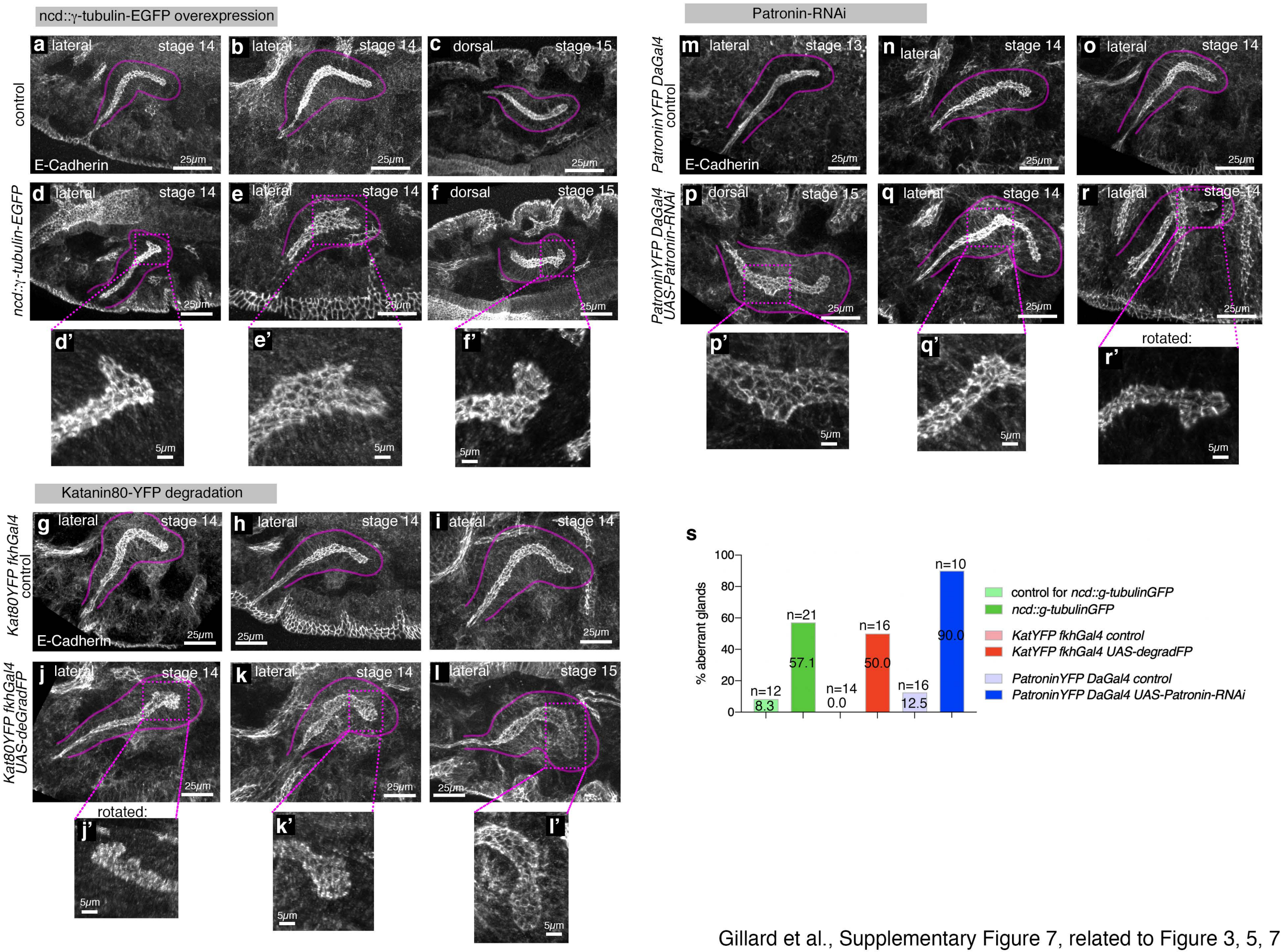

Gillard et al., Supplementary Figure 7, related to Figure 3, 5, 7
