# Supplementary material for: A release-and-capture mechanism generates an essential non-centrosomal microtubule array during tube budding
Source: Nat Commun. 2021 Jul 2;12:4096. doi: 10.1038/s41467-021-24332-0 (PMC8253823; doi:10.1038/s41467-021-24332-0)
Supplement: Supplementary file 3 — Description of Additional Supplementary Files [file 41467_2021_24332_MOESM3_ESM.pdf]

Description of Additional Supplementary Files

File Name: Supplementary Movie 1
Description: **related to Figure 2 d-e.** Time lapse movie of placodal cells in a EB1-GFP Cnn-RFP embryo, the composite and individual channels are shown. Time frames are 0.55 s apart, EB1-GFP is in green, Cnn-RFP in magenta.

File Name: Supplementary Movie 2
Description: **related to Suppl. Fig. S4k-l''.** Time lapse movie of placodal cells in a control embryo of the genotype Asl-GFP EB1-mCherry and in a γ-tubulin-EGFP overexpressing embryo of the genotype ncd::γ-tubulin-EGFP EB1- mCherry. Time frames are 1.14 s apart, Asl-GFP and γ-tubulin-EGFP are in magenta, EB1-mCherry is shown in the inverse channel.

File Name: Supplementary Movie 3
Description**: related to Figure 7i.** Time lapse movie of epidermal cells in a Jupiter-GFP Patronin-RFP embryo, composite and individual channels are shown with JupiterGFP in green and Patronin-RFP in magenta. Between frame 2 and 3 a laser is focused on a subapical area without microtubules in this control movie. Time frames are 1.13 s apart.

File Name: Supplementary Movie 4
Description: **related to Figure 7j.** Time lapse movie of early stage 11 placodal cells in a Jupiter-GFP Patronin-RFP embryo, composite and individual channels are shown with Jupiter-GFP in green and Patronin-RFP in magenta. Between frame 2 and 3 a laser is focused on a microtubule bundle in the apical area to sever it. Time frames are 1.13 s apart.

File Name: Supplementary Movie 5
Description: **related to Figure 7k.** Time lapse movie of stage 12 placodal cells in a Jupiter-GFP Patronin-RFP embryo, composite and individual channels are shown with Jupiter-GFP in green and Patronin-RFP in magenta. Between frame 2 and 3 a laser is focused on the apical region of a longitudinal microtubule bundle to sever it. Time frames are 1.13 s apart.

File Name: Supplementary Movie 6
Description: **related to Figure S5l.** Time lapse movie of a mitotic epidermal cell in a Jupiter-GFP Patronin-RFP embryo, composite and individual channels are shown with Jupiter-GFP in green and Patronin-RFP in magenta. Between frame 2 and 3 a laser is focused on a microtubule bundle within the mitotic spindle to sever it. Time frames are 1.13 s apart.
