# Supplementary material for: A release-and-capture mechanism generates an essential non-centrosomal microtubule array during tube budding
Source: Nat Commun. 2021 Jul 2;12:4096. doi: 10.1038/s41467-021-24332-0 (PMC8253823; doi:10.1038/s41467-021-24332-0)
Supplement: Supplementary file 10 — Reporting Summary [file 41467_2021_24332_MOESM10_ESM.pdf]

nature research

Corresponding author(s): Katja Röper

Last updated by author(s): Apr 25, 2021

Reporting Summary

Nature Research wishes to improve the reproducibility of the work that we publish. This form provides structure for consistency and transparency in reporting. For further information on Nature Research policies, see our Editorial Policies and the Editorial Policy Checklist.

Statistics

For all statistical analyses, confirm that the following items are present in the figure legend, table legend, main text, or Methods section.

n/a	Confirmed	
☐	☒	The exact sample size (n) for each experimental group/condition, given as a discrete number and unit of measurement
☐	☒	A statement on whether measurements were taken from distinct samples or whether the same sample was measured repeatedly
☐	☒	The statistical test(s) used AND whether they are one- or two-sided<br>*Only common tests should be described solely by name; describe more complex techniques in the Methods section.*
☒	☐	A description of all covariates tested
☐	☒	A description of any assumptions or corrections, such as tests of normality and adjustment for multiple comparisons
☐	☒	A full description of the statistical parameters including central tendency (e.g. means) or other basic estimates (e.g. regression coefficient) AND variation (e.g. standard deviation) or associated estimates of uncertainty (e.g. confidence intervals)
☐	☒	For null hypothesis testing, the test statistic (e.g. F, t, r) with confidence intervals, effect sizes, degrees of freedom and P value noted<br>*Give P values as exact values whenever suitable.*
☒	☐	For Bayesian analysis, information on the choice of priors and Markov chain Monte Carlo settings
☒	☐	For hierarchical and complex designs, identification of the appropriate level for tests and full reporting of outcomes
☒	☐	Estimates of effect sizes (e.g. Cohen's d, Pearson's r), indicating how they were calculated

Our web collection on statistics for biologists contains articles on many of the points above.

Software and code

Policy information about availability of computer code

Data collection	Fixed samples were acquired with the Olympus FV10-ASW (v04.02) or Leica LAS-X software for 4-colour imaging. Live imaging was performed with Leica LAS-X or Zeiss Zen 2.1 SP3 (for Patronin-YFP movies) software.
Data analysis	Imaging data were analysed and processed with Fiji 2.0.0 (open source) or Imaris 8.4.1 (Bitplane) for 3D rendering. Cell segmentation and quantification of apical area were performed in otracks (custom software written in IDL, from L3Harris Geospatial (https://www.l3harrisgeospatial.com/Software-Technology/IDL), PMID: 19412170, PMID: 24914560) Statistical analyses were done in GraphPad Prism (v8.4.0), except for the Kolmogorov-Smirnov tests that were performed using Matlab 2018a.

For manuscripts utilizing custom algorithms or software that are central to the research but not yet described in published literature, software must be made available to editors and reviewers. We strongly encourage code deposition in a community repository (e.g. GitHub). See the Nature Research guidelines for submitting code & software for further information.

Data

Policy information about availability of data

All manuscripts must include a data availability statement. This statement should provide the following information, where applicable:

- Accession codes, unique identifiers, or web links for publicly available datasets
- A list of figures that have associated raw data
- A description of any restrictions on data availability

The authors declare that all relevant data are included in the paper or the supplementary information files.

Field-specific reporting

Please select the one below that is the best fit for your research. If you are not sure, read the appropriate sections before making your selection.

- [x] Life sciences
- [ ] Behavioural & social sciences
- [ ] Ecological, evolutionary & environmental sciences

For a reference copy of the document with all sections, see nature.com/documents/nr-reporting-summary-flat.pdf

Life sciences study design

All studies must disclose on these points even when the disclosure is negative.

Sample size	Sample size was not predetermined by any statistical method but rather based on previous studies in the field (see for example PMID: 32483386; PMID: 31227595; PMID: 31805038; PMID: 31353316; PMID: 28988857). All information about sample size (embryos, cells, centrosomes, junctions) can be found next to the graphs, in the legends and in the methods section of the paper.
Data exclusions	No embryos were excluded from the analysis except in Spastin overexpression experiments, where embryos without a reduction in the α-acetylated tubulin staining were excluded.
Replication	All experiments were repeated at least twice. Consistency across replicates is captured in each experiment by means and standard deviations calculated across multiple replicates. All attempts at replication were successful to the extent reflected in means, data distributions and statistical tests described in the paper.
Randomization	The experiments were not randomized, and the investigators were not blinded to allocation during experiments and outcome assessment. Automatic image analysis provided an objective and unbiased evaluation of phenotypes in most experiments.
Blinding	The experiments were not randomized, and the investigators were not blinded to allocation during experiments and outcome assessment. Blinding was not relevant to this study because experimental procedures did not allow randomization. Additionally, automatic image analysis provided an objective and unbiased quantification of apical areas and apical-medial accumulation of given markers.

Reporting for specific materials, systems and methods

We require information from authors about some types of materials, experimental systems and methods used in many studies. Here, indicate whether each material, system or method listed is relevant to your study. If you are not sure if a list item applies to your research, read the appropriate section before selecting a response.

Materials & experimental systems

n/a	Involved in the study	
[ ]	[x]	Antibodies
[x]	[ ]	Eukaryotic cell lines
[x]	[ ]	Palaeontology and archaeology
[ ]	[x]	Animals and other organisms
[x]	[ ]	Human research participants
[x]	[ ]	Clinical data
[x]	[ ]	Dual use research of concern

Methods

n/a	Involved in the study	
[x]	[ ]	ChIP-seq
[x]	[ ]	Flow cytometry
[x]	[ ]	MRI-based neuroimaging

Antibodies

Antibodies used

Primary antibodies:
- Rabbit polyclonal anti-CrebA (CrebA Rbt-PC, dilution 1:1000), Mouse monoclonal anti-Crumbs (Cq4, dilution 1:10) and Rat monoclonal anti-E-cadherin (DCAD2, dilution 1:10) were obtained from the Developmental Studies Hybridoma Bank at the University of Iowa.
- Rabbit polyclonal anti-aPKC was from Santa Cruz Biotech (sc-216, dilution 1:1000).
- Mouse monoclonal anti-γ-tubulin (GTU-88, Cat#T6557, dilution 1:500), Mouse monoclonal anti-acetylated α-tubulin (6-11B-1, Cat#T7451, dilution 1:500) and Mouse monoclonal anti-α-tubulin (DM1A, Cat#T9026, dilution 1:1000) were from Sigma.
- Rabbit polyclonal anti-phospho-Histone H3 [Ser10] (#9701, dilution 1:500) was from Cell Signalling Technology.
- Rat polyclonal anti-tyrosinated α-tubulin YL1/2 (described in ref [64], dilution 1:10) is a gift from John Kilmartin.
- Rabbit polyclonal anti-AslNT (described in ref [66], dilution 1:1000) and Rabbit polyclonal anti-Cnn (described in ref [65], dilution 1:000) were gifts from Jordan Raff, Sir William Dunn School of Pathology, University of Oxford, UK.
- Guinea pig polyclonal anti-Shot was previously made in the lab and was described in ref [67] (dilution 1:1000).

Secondary antibodies: All used at 1:200 dilution.
1) From Jackson Immunoresearch:
- Alexa Fluor® 488 AffiniPure Donkey Anti-Rabbit IgG (H+L) (711-545-152); Cy™3 AffiniPure Donkey Anti-Rabbit IgG (H+L)

(711-165-152); Alexa Fluor® 647 AffiniPure Donkey Anti-Rabbit IgG (H+L) (711-605-152).
- Cy™3 AffiniPure Donkey Anti-Mouse IgG (H+L) (715-165-151); Cy™5 AffiniPure Donkey Anti-Mouse IgG (H+L) (715-175-151).
- Cy™3 AffiniPure Goat Anti-Rat IgG (H+L) (112-165-167); Alexa Fluor® 647 AffiniPure Donkey Anti-Rat IgG (H+L) (712-605-153).
- Cy™5 AffiniPure Donkey Anti-Guinea Pig IgG (H+L) (706-175-148).
2) From Invitrogen:
- Donkey anti-Rabbit IgG (H+L) Highly Cross-Adsorbed Secondary Antibody, Alexa Fluor Plus 405 (A48258).
- Goat anti-Mouse IgG (H+L) Highly Cross-Adsorbed Secondary Antibody, Alexa Fluor 350 (A-21049); Donkey anti-Mouse IgG (H+L) Highly Cross-Adsorbed Secondary Antibody, Alexa Fluor Plus 488 (A32766).
- Goat anti-Rat IgG (H+L) Cross-Adsorbed Secondary Antibody, Alexa Fluor 488 (A-11006).
Rhodamine-phalloidin was from Thermofisher (R415, dilution 1:500).

Validation

All antibodies used in this study have previously been validated in other studies or by the manufacturer, as per statement below and were validated by us by comparing our results to well characterized distribution of the markers.
- The anti-CrebA, anti-Crumbs and anti-E-cadherin have been validated by depositors. The anti-CrebA expression reflects its specific localization in the salivary gland placode at stage 11 while anti-Crumbs and anti-E-Cadherin label cell membranes as described in the literature.
The anti-Dcad2 was deposited to the DSHB by T. Uemura and was validated by immunoblotting and immunoprecipitation experiments using 0- to 15-hour embryos as compared to an anti-mouse E-cadherin monoclonal antibody ECCD-2 (PMID: 7958432).
The anti-CrebA was deposited to the DSHB by D. Andrew and was validated in Drosophila embryos by immunofluorescent staining where it gave identical staining patterns in embryos that match the accumulation pattern of dCREB-A transcripts (PMID: 9006079).
The anti-Crumbs was deposited to the DSHB by E. Knust and was validated by Western blot using the same part of the CRB proteins that was used for immunisation as well as by wholemount staining of wild type embryos (PMID: 8365569).
- The anti-aPKC antibody has been validated by Western Blot by the manufacturer (https://www.scbt.com/p/pkc-zeta-antibody-c-20?requestFrom=search) and also label cell membranes as expected.
- All antibodies from Sigma (anti-α-tubulin: https://www.sigmaaldrich.com/catalog/product/sigma/t9026?lang=en&region=GB; anti-acetylated α-tubulin: https://www.sigmaaldrich.com/catalog/product/sigma/t7451?lang=en&region=GB; anti-γ-tubulin; https://www.sigmaaldrich.com/catalog/product/sigma/t6557?lang=en&region=GB) have been validated by the manufacturer by Western Blot and immunofluorescence where they exhibit the appropriate localization pattern as reported in the literature.
- The anti-phospho-histone H3 has been validated by Western Blot by the manufacturer (https://www.cellsignal.com/products/primary-antibodies/phospho-histone-h3-ser10-antibody/9701).
- The anti-AslNT (aa 1-333) and anti-Cnn have been affinity purified and validated by immunofluorescent staining (PMID: 19948479 and PMID: respectively) against the well-characterized expression pattern of the genes documented in the literature.
- The anti-tyrosinated α-tubulin YL1/2 has been first characterized through binding assays with yeast and chick brain tubulin, then through immunofluorescent staining of mitotic spindles in yeast and the interphase network of microtubules in CHO and 3T cells (PMID: 6811596).
- Finally, the anti-Shot has been characterized in Western blots of whole Drosophila embryo lysates as compared to a second antibody targeting another domain of Shot and immunofluorescent staining in Drosophila embryos (PMID: 14517208).
All anti-tubulin antibodies were further validated in Drosophila embryos in our lab with a loss of staining upon microtubule depletion following Spastin overexpression.

Animals and other organisms

Policy information about studies involving animals; ARRIVE guidelines recommended for reporting animal research

Laboratory animals

Drosophila melanogaster embryos were used in this study, regardless of their sex. Drosophila melanogaster is not classified as an animal under current legislation in both the UK and the US. Stage 10-11 and stage 16 embryos were imaged and analysed. Those were obtained 6 to 8 hours or16 hours after egg-laying at 25 degrees to capture the process of apical constriction and tissue invagination in the salivary gland placode.
The following fly stocks were used in this study: from Bloomington Stock Centre: Daughterless-Gal4 (Da-Gal4; #27608); UAS-Patronin-RNAi (y1 sc* v1; P{TRiP.HMS01547}attP2) #36659); ncd-γ-tubulin-EGFP (w1118; P{ncd-γTub37C.GFP}F13F3)(#56831). Furthermore Katanin80-YFP (w1118 PBac{602.P.SVS-1}kat80CPTI000764) [Kyoto Stock Centre; CPTI 000764]; fkh-Gal4 on chromosome III (refs 53, 54; Asterless-GFP on X, Ubi-EB1-mCherry on X and Asterless-mCherry (w; eAsl-mch/Cyo; MKRS/TM6b) [gifts form Jordan Raff, Sir William Dunn School of Pathology, University of Oxford, UK]; YFP-Cnb (w1118; pUbi-YFP-Cnb) (ref. 29); RFP-Cnn (ref. 30); Ubi-EB1-GFP (ref. 55); Patronin-RFP (Patronin-TagRFPattp40[22H02-C]) (ref. 56); Patronin-YFP (w1118; Patronin-YFP/Cyo) (ref. 18); GFP-Polo (ref. 57); Sas-4-GFP (ref. 58); Scribble-GFP (w; P{PTT-GA}scribCA07683) (ref. 59); Spd-2-GFP (ref. 60); sqh-TagRFPt[9B] (ref. 33); UAS-deGradFP (w; If/Cyo; UAS>NSlmb-vhhGFP4/TM6b) (ref. 40); UAS-Spastin on X (ref. 61); Jupiter-GFP (P{PTT-GA}JupiterG00147) (ref. 62); fkh-Gal4 UAS-srcGFP (ref. 63).
The following combinations of transgenes were generated in this study through genetic crosses:
Asl-mCherry; Jupiter-GFP
EB1-mCherry; ; ncd::gamma-tubulin-EGFP
sqh-TagRFPt[9B]; ; ncd::gamma-tubulin-EGFP
Katanin80-YFP; Asl-mCherry
Katanin80-YFP; ; fkh-Gal4
Katanin80-YFP; ; UAS-degradFP
Katanin80-YFP; Patronin-RFP; fkh-Gal4
Katanin80-YFP; Patronin-RFP; UAS-degradFP
Patronin-RFP; ncd::gamma-tubulin-EGFP
Patronin-RFP; Jupiter-GFP
Patronin-YFP; Da-Gal4
Patronin-YFP; fkh-Gal4
Patronin-YFP; UAS-degradFP

	UAS-spastin; Patronin-YFP<br>Patronin-YFP; Ubi-TagRFP
Wild animals	This study did not involve wild animals.
Field-collected samples	This study did not involved samples collected from the field.
Ethics oversight	This study did not require ethics oversight.

Note that full information on the approval of the study protocol must also be provided in the manuscript.
